# Freehand Ventriculoperitoneal Shunt for Idiopathic Intracranial Hypertension: Technical Note for Slit-Like Ventricle Puncture

**DOI:** 10.7759/cureus.34583

**Published:** 2023-02-03

**Authors:** José Orlando de Melo Junior, Paulo José da Mata Pereira, Paulo Niemeyer Filho

**Affiliations:** 1 Department of Neurosurgery, Paulo Niemeyer State Brain Institute, Rio de Janeiro, BRA; 2 Department of Neurosurgery, Santa Teresa Hospital, Petrópolis, BRA

**Keywords:** ventriculoperitoneal shunt placement, small ventricle, slit-like ventricle, idiopathic intracranial hypertension, freehand technique

## Abstract

Idiopathic intracranial hypertension (IIH) is a syndrome characterized by elevated intracranial pressure, headache, and papilledema. It is frequently associated with obese women and can result in irreversible vision loss. The ventriculoperitoneal (VP) shunt has been proven to be superior to the lumboperitoneal (LP) shunt in IIH patients, with better clinical outcomes. It has been reported that the accurate placement of the ventricular catheter is highly important for shunt survival. However, a slit-like ventricle pattern, typically seen in the disease, has been considered a great concern and challenge for ventricular catheter placement, primarily for freehand technique. Frameless stereotaxy, ultrasound, and endoscopy have been described to improve the accuracy of catheter insertion. However, intraoperative image guidance is not widely accessible, especially in lower-resource countries, due to the high costs associated with its use. Techniques to improve the accuracy of the freehand VP shunt in IIH are scarce in the literature, and any effort to contribute to its development is valuable and helpful.

## Introduction

Idiopathic intracranial hypertension (IIH) is a syndrome characterized by elevated intracranial pressure, headache, and papilledema. It is frequently associated with obese women and can result in irreversible vision loss [1].

Indications for surgical intervention include patients with disease clinically refractory to medication and rapid deterioration of vision [2]. Historically, the traditional treatment has been the placement of a lumboperitoneal (LP) shunt, however, a ventriculoperitoneal (VP) shunt has been proven to be superior in IIH patients in terms of revision surgery [3].

Accurate ventricular catheter placement plays an important role in reducing the risk of VP shunt failure [4]. IIH patients typically have very small-size ventricles (slit-like ventricles), which imposes a great concern and challenge for freehand ventricular catheter placement [3]. Techniques to improve a freehand VP shunt in IIH are scarce in the literature.

Image guidance can be used to ensure accurate ventricular catheter placement [5]. However, these technologies have been associated with additional operative time and cost and are often not available worldwide [6,7]. Thus, any effort to improve the accuracy of the freehand technique for ventricular catheter placement in IIH patients is valuable and helpful, especially for developing countries with restricted use of equipment and technologies.

## Technical report

Two cases of IIH patients treated with a freehand VP shunt with a fixed medium-pressure valve system are presented, using an intuitive radiological reference technique for successful ventricular catheter placement.

Case 1

**Figure 1:**
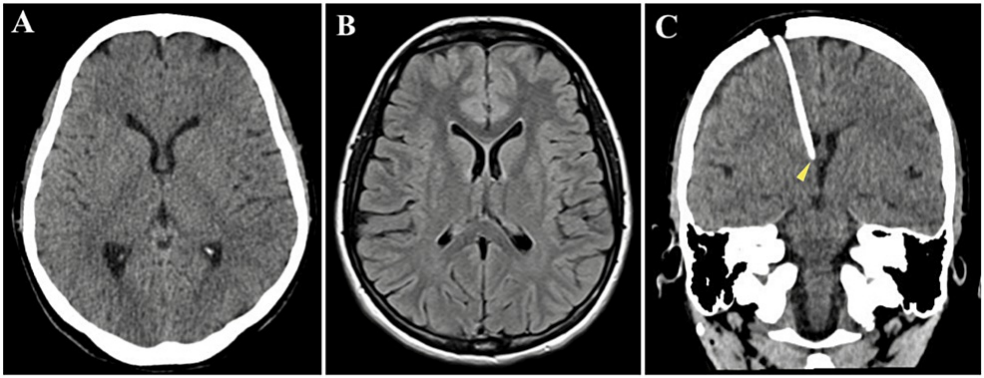
Case 1 Axial brain CT scan (A) and MRI (B) show a slit-like ventricle pattern. (C) Postoperative coronal CT scan shows accurate ventricular catheter position in a sufficient pool of CSF with the tip in the foramen of Monro (yellow arrowhead).

A 21-year-old female patient presented with a history of headache, nausea, vomiting, and blurred vision in the last five months. An ophthalmological examination demonstrated bilateral papilloedema. Imaging studies ruled out structural and obstructive lesions as possible causes of her symptoms, and lumbar puncture results were unremarkable except for an increased opening pressure of 36 cmH2O. Brain computed tomography (CT) scan and magnetic resonance imaging (MRI) showed a slit-like ventricle pattern (Figure 1A and Figure 1B). She had been treated clinically with acetazolamide, topiramate, and serial lumbar punctures by a neurologist without consistent improvement in symptoms, which eventually resulted in worsening and severe loss of visual acuity. She was then referred to our center for surgical treatment of medically refractory IIH. The patient was then submitted to a freehand VP shunt with a fixed medium-pressure valve system. Ventricular catheter placement was achieved in a single passe. A postoperative brain CT scan on the next day revealed accurate catheter placement (Figure 1C). At the follow-up, the patient had a good postoperative outcome with a resolution of symptoms and improvement in visual acuity.

Case 2

**Figure 2:**
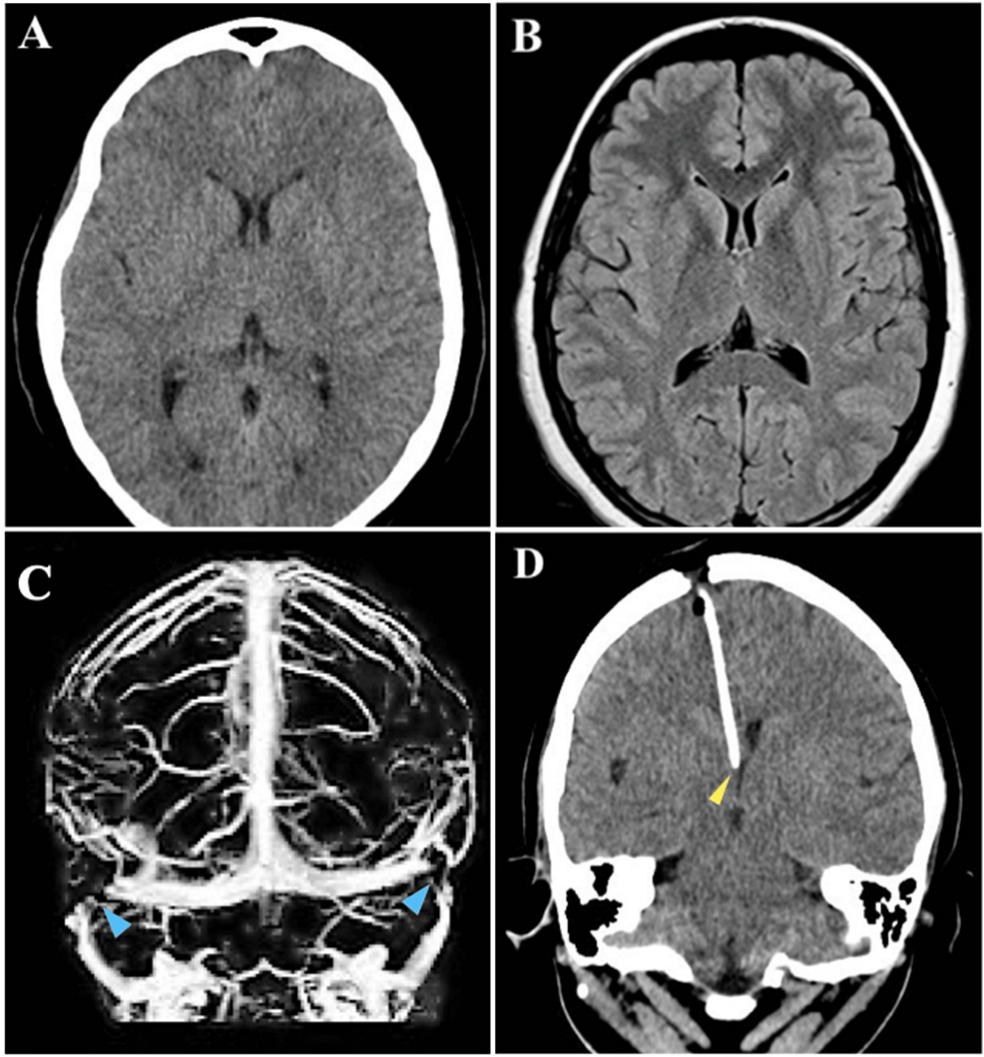
Case 2 Axial brain CT scan (A) and MRI (B) show a slit-like ventricle pattern. (C) Brain MRI venous angiography shows bilateral sinus stenosis between the transverse and sigmoid sinuses (blue arrowheads). (D) Postoperative coronal CT scan shows accurate ventricular catheter position in a sufficient pool of CSF with the tip in the foramen of Monro (yellow arrowhead).

A 25-year-old female patient was referred to our center with a history of IIH refractory to medical treatment with acetazolamide, topiramate, serial lumbar punctures, and weight loss. She presented symptoms of headache and visual deterioration in the last year. Ophthalmological examination demonstrated bilateral papilloedema and lumbar puncture showed increased opening pressure of 36 cmH2O. Brain CT scan and MRI showed a slit-like ventricle pattern (Figure 2A and Figure 2B). Brain MRI venous angiography demonstrated bilateral transverse sinus stenosis (Figure 2C). A venous sinus stenting was attempted with the endovascular team. However, it was not possible due to the patient’s inherited anatomical limitations. Therefore, a VP shunt with a fixed medium pressure valve system was carried out for definite treatment. Ventricular catheter placement was achieved in a single pass. A postoperative brain CT scan on the next day revealed accurate catheter placement (Figure 2D). At the follow-up, the patient had a good postoperative outcome with the resolution of headache and improvement in visual acuity.

Modified Kocher’s point for a slit-like ventricle

**Figure 3:**
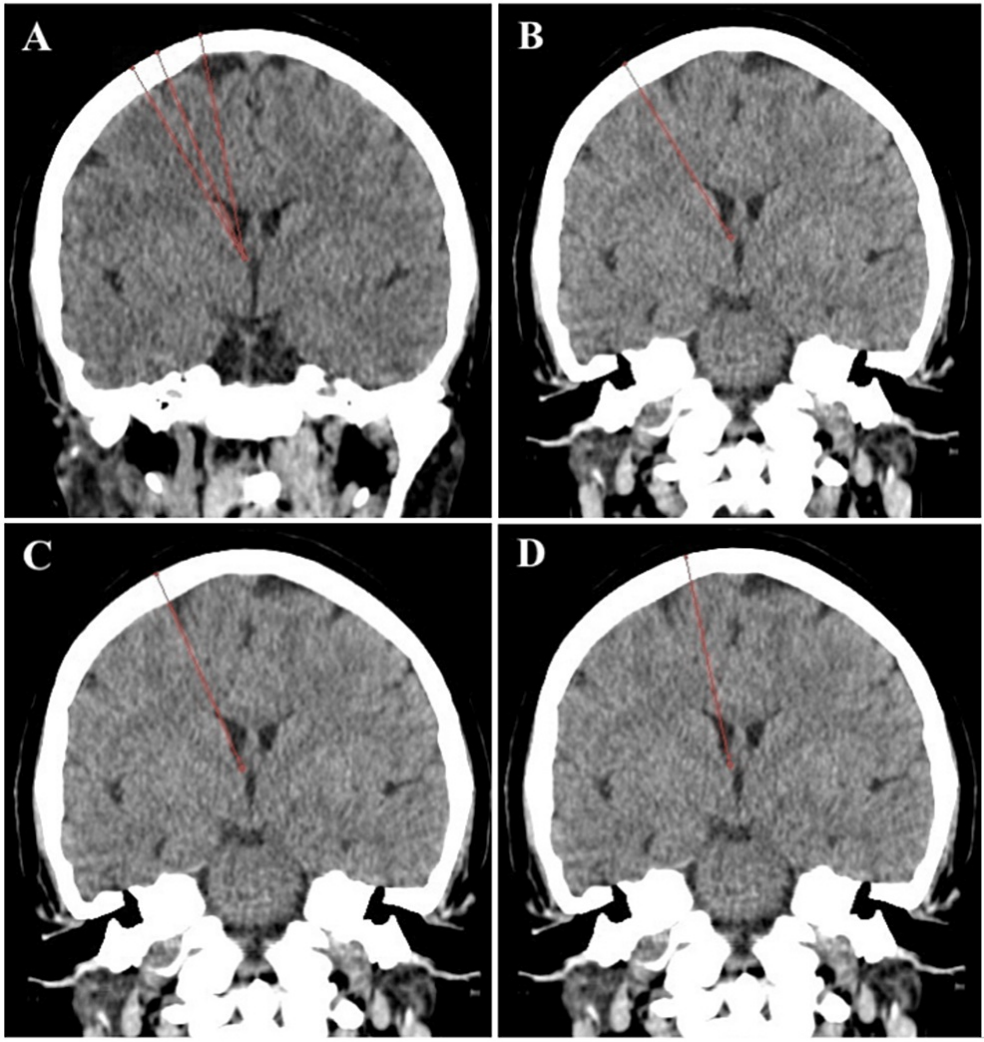
Possible catheter trajectories (A) Trajectories (red lines) through Kocher’s entry point at different distances from the midline to the foramen of Monro. (B) The trajectory through an entry point 4 cm from the midline. Note that the trajectory crosses the head of the caudate nucleus and most parts of the catheter would be outside the ventricle (red line). (C) The trajectory through an entry point 3 cm from the midline. Note that the trajectory is tangent to the ventricular wall (red line). (D) The trajectory through an entry point 1.5 cm from the midline. Note that the trajectory is perpendicular to the roof of the frontal horn and traverses a long pool of CSF to the foramen of Monro (red line).

**Figure 4:**
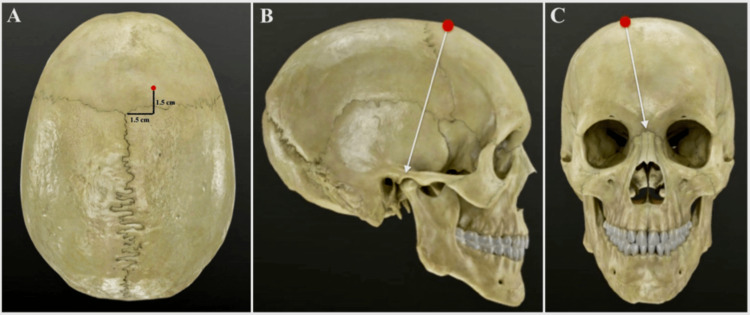
Modified Kocher’s point for a slit-like ventricle (A) A modified Kocher’s entry point for a slit-like ventricle. The entry point (red dot) is located 1.5 cm anterior to the bregma, or 11.5 cm behind the nasion, and 1.5 cm lateral to the midline. (B) Entry point (red dot) and anteroposterior inclination (white line) toward the tragus. (C) Entry point (red dot) and medial inclination (white line) towards the nasion.

A modified Kocher’s point for a slit-like ventricle is placed at 11.5 cm behind the nasion, or 1.5 cm anterior to the bregma, and 1.5 cm lateral to the midline, preferably on the right side (Figure 3A). This point is placed more medially than usual and therefore may increase the risk of venous injury. Hence, a careful radiological examination, which includes a brain MRI, should rule out a significant venous lake or large vein crossing the surgical site. The length of the catheter that will be inserted is also measured on a preoperative coronal CT scan from the outer table of the skull to the foramen of Monro. This medialization of the entry point has the advantage of preventing the catheter from being tangent to the ventricular wall or even passing through the basal ganglia and internal capsule (Figure 4). Furthermore, it provides the opportunity to insert the catheter in a long pool of CSF.

Surgical technique

The skin is marked over the planned entry point on the scalp with a surgical pen. The patient is placed in the supine position and the head is rotated to the contralateral side. The neck and trunk are extended with the assistance of a roll under the shoulders. Local trichotomy is then proceeded. The surgical field is prepared and draped in a sterile fashion. It is important to ensure that the surgical drapes in the cranial region are properly secured with skin sutures to avoid contamination during head rotation, as described below.

The head and abdominal surgical fields are started together by two different neurosurgeons. A right frontal longitudinal paramedian skin incision is performed long enough to make a burr hole. The dura is opened. The abdominal skin incision is carried out, usually in the right upper quadrant, and the peritoneal cavity is opened. The distal catheter is tunneled subcutaneously connecting the abdomen to a small incision in the parietal region. The distal catheter is then tunneled from the parietal to the frontal incision and then connected to a subcutaneously placed valve. Next, the head is rotated and moved to a neutral position to improve the surgeon’s spatial orientation and to increase the chance of a successful frontal horn puncture. The ventricular catheter must have two coordinates, the anteroposterior and laterolateral angulations. The anteroposterior angulation is obtained by pointing the catheter tip towards the tragus (Figure 3B). The laterolateral angulation is obtained by pointing the catheter tip to the nasion (Figure 3C). The ventricular catheter is then inserted into the frontal horn of the lateral ventricle. The length of the inserted catheter is guaranteed, as planned preoperatively, and connected to the valve. Proper flow of CSF through the peritoneal catheter is certified before being inserted into the peritoneal cavity. Finally, abdominal and cranial sites are closed in a standard fashion. A postoperative brain CT scan on the next day is performed to verify the proper placement of the ventricular catheter.

## Discussion

IIH is a clinical condition characterized by an increase in intracranial pressure, headache, papilledema, and vision loss in the absence of any identifiable causal factor [7]. Some causes of secondary intracranial hypertension are depicted in Table 1 [2]. The modified Dandy diagnostic criteria are (a) papilledema or abducens palsy, (b) increased lumbar puncture opening pressure (≥ 250 mmH20 in adults) with normal CSF constitution, (c) normal neurological examination except for cranial nerve abnormalities, and (d) brain imaging with no evidence of pathologies such as hydrocephalus, structural lesion or abnormal meningeal enhancement [1].

**Table 1:** Some causes of secondary intracranial hypertension

Group	Causes
Vascular	Dural venous sinus thrombosis, bilateral jugular vein thrombosis or surgical ligation, bilateral transverse sinus stenosis, central venous hypertension due to heart failure, arteriovenous malformations or dural fistulae with high flow, superior vena cava syndrome
Endocrine	Addison disease, hypoparathyroidism, hormones exposure (human growth hormone, thyroxine, levonorgestrel, anabolic steroids), withdrawal from chronic corticosteroids
Drug	Tetracycline, doxycycline, lithium
Genetic	Turner syndrome, Down syndrome
Others	Previous meningitis or subarachnoid hemorrhage, retinoids exposure and hypervitaminosis A, renal failure, sleep apnea

Clinical treatment with acetazolamide, topiramate, and weight loss are indicated as the first-line treatment, with approximately 75% of patients being effectively controlled [2]. Indications for surgical intervention are patients with clinically refractory disease and rapid deterioration of vision, and surgical procedures include optic nerve sheath fenestration, CSF diversion (LP or VP shunts), venous sinus stenting and bariatric surgery, with no controlled trials comparing them [2].

An LP shunt is classically the first-line surgical procedure for patients with IIH, despite the lack of prospective randomized studies [8]. However, VP shunt placement has been associated with better clinical outcomes with less revision surgery, mean hospital length of stay, as well as burdens on the health care system [3]. Although being a relatively easier, faster, and safer technique, LP shunt failure and revision rates were found to be unacceptably high in some studies, most of which were related to the migration of peritoneal catheters [9].

The typical concern and aversion of VP shunt in IIH has been imputed to the typically slit-like ventricles of the disease [3]. The literature shows that patients with the highest number of inaccurately placed ventriculostomies are patients with smaller ventricles [10]. The insertion of a ventricular catheter, in such cases, has been considered technically challenging, and this may necessitate a stereotactic insertion, which is time-consuming, expensive, and not widely available [11]. A retrospective study of freehand VP shunts has shown that a ventricular catheter was less than optimally placed in 26.8%, almost 10% outside the ventricular system, with the most important predictors being younger age and smaller ventricular size [4].

VP shunt malfunction is often caused by obstruction, particularly at the ventricular catheter [12,13]. Hence, accurate placement is one of the most important predictors of shunt survival [4]. Ginsberg et al. showed that the resistance of CSF drainage was inversely proportional to the number of patent holes in the ventricular catheter [14]. Tuli et al. supported that a ventricular catheter tip surrounded by CSF was associated with a decreased risk of shunt failure [13]. Thomale et al. also assumed that in narrow ventricles the catheter perforations that are located in the cerebral tissue might be a risk for CSF shunt obstruction [15]. Along these lines, the main goal in ventricular catheter insertion is to place it in a sufficient pool of CSF to avoid obstruction of the catheter’s perforation holes by the brain parenchyma, with precise positioning of the tip.

A VP shunt is performed using standard cranial points, depending upon the entry site, such as Frazier's point for the occipital, Keen’s for the parietal, and Kocher's for the frontal [16]. However, there is conflicting evidence on the best approach and method for a VP shunt [4]. The frontal approach has the shortest route with more consistent anatomical landmarks [17], and the occipital approach has a worse outcome with a smaller margin of error for trajectories [4]. The frontal approach is also one of the most commonly performed neurosurgical procedures, often used in external ventricular drain (EVD) placement [18]. Lind et al. showed that it provides the largest margin of error for selecting the lateral ventricle catheter trajectory in normal and hydrocephalic ventricles [17]. We believe that the frontal approach is the most logical in terms of ventricular catheter accuracy for IIH without image guidance, with the unique disadvantage of the need for an extra tunneling incision to reach this location from the abdomen. The ideal goal for the frontal approach involves the ventricular catheter ending within the ipsilateral frontal horn of the lateral ventricle or in the third ventricle and has been associated with better long-term VP shunt patency [7,10].

Several different entry points for the frontal approach have been described in the literature and are often called Kocher’s point. The entry points vary from 1.5 to 4 cm lateral to the midline and from 10 to 12.5 cm behind the nasion, and trajectories in the coronal plane are toward the ipsilateral medial canthus, nasion, or contralateral medial canthus, and in the sagittal plane are “downward and backward”, through the external acoustic meatus, tragus, or 1-1.5 cm anterior to the tragus [19]. Raabe et al. showed that entry points 1 or 2 cm lateral to the midline had the highest rate of hitting the center of the anterior horn of the lateral ventricle when combined with a trajectory toward the nasion, and 3 or 4 cm lateral to the midline only in combination with a trajectory toward the contralateral canthus [19]. However, their study did not evaluate patients with slit-like ventricles.

The freehand technique using surface anatomical landmarks for VP shunts has been shown to be less accurate over ultrasonic guidance and stereotactic neuronavigation for ventricular catheter placement, with increased proximal failures [11]. Previous studies have reported a low success rate of the frontal approach with the freehand technique, ranging from 43.9% to 64% [20]. Nevertheless, techniques to improve freehand VP shunts in IIH are scarce in the literature and any effort to contribute to its development is valuable and helpful.

To overcome significant catheter misplacements, several expensive alternative methods that are unfortunately not available worldwide have been used [6]. Intraoperative imaging guidance has been employed to overcome the challenge of the slit-like ventricle in IIH and includes the use of stereotaxis, ultrasound, and endoscopy [7]. Image-guided frameless stereotaxis has allowed the procedure to be performed safely and with great accuracy, with only one pass of the catheter needed on each patient [5]. Nevertheless, frameless stereotaxy has some disadvantages, including the need for head fixation and physical limitations on positioning often obese patients, higher cost, longer surgical time, and limited accessibility in countries with few resources [7].

## Conclusions

Techniques to improve the accuracy of freehand VP shunt in IIH are scarce in the literature and any effort to contribute to its development is valuable and helpful since intraoperative image guidance is not widely accessible, especially in lower-resource countries, due to the high costs associated with its use. Further larger studies are needed to evaluate whether the described technique is a reliable method to improve the accuracy of freehand ventricular catheter placement in the slit-like ventricles of IIH.
